# Potential Application of *Apilactobacillus kunkeei* for Human Use: Evaluation of Probiotic and Functional Properties

**DOI:** 10.3390/foods9111535

**Published:** 2020-10-25

**Authors:** Franca Vergalito, Bruno Testa, Autilia Cozzolino, Francesco Letizia, Mariantonietta Succi, Silvia Jane Lombardi, Patrizio Tremonte, Gianfranco Pannella, Roberto Di Marco, Elena Sorrentino, Raffaele Coppola, Massimo Iorizzo

**Affiliations:** 1 Department of Agricultural, Environmental and Food Sciences (DiAAA), University of Molise, via De Sanctis snc, 86100 Campobasso, Italy; franca.vergalito@unimol.it (F.V.); bruno.testa@unimol.it (B.T.); a.cozzolino@studenti.unimol.it (A.C.); francesco.letizia@unimol.it (F.L.); silvia.lombardi@unimol.it (S.J.L.); tremonte@unimol.it (P.T.); gianfranco.pannella@unimol.it (G.P.); sorrentino@unimol.it (E.S.); coppola@unimol.it (R.C.); iorizzo@unimol.it (M.I.); 2 Department of Medicine and Health Sciences “Vincenzo Tiberio”, University of Molise, via De Sanctis snc, 86100 Campobasso, Italy; roberto.dimarco@unimol.it

**Keywords:** lactobacilli, fructophilic lactic acid bacteria, antibiotic susceptibility, antimicrobial activity, gastrointestinal transit, probiotication

## Abstract

*Apilactobacillus kunkeei* is an insect symbiont with documented beneficial effects on the health of honeybees. It belongs to fructophilic lactic acid bacteria (FLAB), a subgroup of lactic acid bacteria (LAB) notably recognized for their safe status. This fact, together with its recurrent isolation from hive products that are traditionally part of the human diet, suggests its possible safe use as human probiotic. Our data concerning three strains of *A. kunkeei* isolated from bee bread and honeybee gut highlighted several interesting features, such as the presence of beneficial enzymes (β-glucosidase, β-galactosidase and leucine arylamidase), the low antibiotic resistance, the ability to inhibit *P. aeruginosa* and, for one tested strain, *E. faecalis*, and an excellent viability in presence of high sugar concentrations, especially for one strain tested in sugar syrup stored at 4 °C for 30 d. This datum is particularly stimulating, since it demonstrates that selected strains of *A. kunkeei* can be used for the probiotication of fruit preparations, which are often used in the diet of hospitalized and immunocompromised patients. Finally, we tested for the first time the survival of strains belonging to the species *A. kunkeei* during simulated gastrointestinal transit, detecting a similar if not a better performance than that showed by *Lacticaseibacillus rhamnosus* GG, used as probiotic control in each trial.

## 1. Introduction

For many years, fermented dairy products were considered as the main source for the isolation of probiotic bacteria [1]. Moreover, various milk-based products such as yoghurt, cheeses and sour milk were primarily used for probiotic incorporation to give microorganisms a protective matrix during their journey into the gastrointestinal tract.

Over the past decades, the scientific research has been increasingly encouraged to select new probiotics from “unconventional sources” [2]. Thus, potentially beneficial bacteria were progressively isolated from numerous non-dairy sources, including meat, fruit, vegetables, cereals and honeybee and beehive products [3,4,5,6,7,8].

The selection process of new potential probiotic strains should focus primarily on their safety, functional properties and beneficial effects on the health of the hosts, rather than on their “origin” [9], intended as the matrix of isolation as well as whether the strains belong to specific genera and species universally recognized as “safe”. For this last aspect, in 2014, the International Scientific Association for Probiotics and Prebiotics (ISAPP) provided a document to clarify the use of the term “probiotic”, including under this definition some bacteria of human gut origin, such as *Akkermansia muciniphila*, *Faecalibacterium prausnitzii*, *Roseburia* spp. and *Eubacterium hallii*, which clearly do not fall in the core group of well-studied species able to confer some general benefits (*Bifidobacterium adolescentis*, *B. animalis*, *B. bifidum*, *B. breve*, *B. longum* and *Lactobacillus acidophilus*, *L. casei*, *L. fermentum*, *L. gasseri*, *L. johnsonii*, *L. paracasei*, *L. plantarum*, *L. rhamnosus* and *L. salivarius*) and generally recognized as safe [10]. In this regard, in the last few years another group of microorganisms, named fructophilic lactic acid bacteria (FLAB), has attracted much attention thanks to their potentially beneficial properties for human health and for their proximity to lactic acid bacteria (LAB), which are, as stated, commonly accepted as safe [11]. FLAB is a specific subgroup of LAB that has been characterized and described only recently [12]. They live in symbiosis with insects that have special diets, such as honeybees [13,14]. In fact, FLAB prefer fructose as growth substrate and they inhabit only fructose-rich niches [12,15], explaining why they are found in different vegetal food matrices such as grapes, durian fruit, figs, banana, cocoa beans and legumes [16].

Among FLAB, there are members of the genus *Lactobacillus* that were recently reclassified as *Apilactobacillus* to underline the specific adaptation to bees [17]. The genus *Apilactobacillus* comprises different species; among them, *A. kunkeei* (basonym *Lactobacillus kunkeei*) and *A. apinorum* (basonym *Lactobacillus apinorum*) have adapted to bees, with niches including honeybees (*Apis mellifera*) and flowers.

*A. kunkeei* is an important component of the gut microbiota of honeybees [18], suggesting that this bacterium may have probiotic functions for these insects.

In fact, *A. kunkeei* displays a broad spectrum of inhibition versus honeybee pathogens, and recent studies have shown that its genome encodes proteins potentially involved in biofilm formation or able to act as antimicrobial compounds against pathogens causing human wound infections [19,20].

Strains of *A. kunkeei* also showed antibiofilm formation properties against virulent *Pseudomonas aeruginosa* strains in vitro and in an insect infection model [21]. In addition, certain extracellular polymeric substances (EPS) produced by *A. kunkeei* are thought to have beneficial effects on human health, such as cholesterol-lowering ability [8].

Among other interesting features of this species, the ability of *A. kunkeei* to metabolize fructose deserves particular interest, because it could ameliorate fructose-mediated irritable bowel syndrome (IBS) [16]. Moreover, by converting fructans into fructose and metabolizing the resulting fructose, these bacteria are able to lower the fermentable oligosaccharides, disaccharides, monosaccharides and polyols (FODMAP) cut-off levels in fruit, vegetables and cereals.

With regard to the safety of this species, it is clear that further studies are required to investigate if these bacteria have a potential use as probiotics in humans. However, it should be considered that *A. kunkeei*, which lives in symbiosis with honeybees, is present in many hive products (honey, royal jelly, bee bread) that have been part of the human diet for hundreds of years. Additionally, there is no clinical evidence regarding the pathogenicity of *A. kunkeei*. Finally, two licenses were already deposited in USA and in Canada for the use of *A. kunkeei* in food and parapharmaceutical products [22,23].

Based on these important observations, in this work we decided to contribute to a greater knowledge on some functional and probiotic properties of *A. kunkeei* strains isolated from bee bread and the gut microbiota of honeybees (*A. mellifera* L.).

## 2. Materials and Methods

### 2.1. Bacterial Strains

The microorganisms used in this study were *Apilactobacillus kunkeei* K18, K34 and K45, isolated from bee bread and the gut microbiota of honeybees (*A. mellifera* L.) and belonging to the DiAAA collection (Department of Agricultural, Environmental and Food Science, University of Molise); *Lacticaseibacillus rhamnosus* GG ATCC 53103, *Pseudomonas aeruginosa* ATCC 27853, *Enterococcus faecalis* ATCC 29212, *Escherichia coli* ATCC 11775, *E. coli* ATCC 25404 and *Staphylococcus aureus* ATCC 29213, belonging to the American Type Culture Collection (ATCC Manassas, VA, USA); and *A. kunkeei* DSM 12361, belonging to the Leibniz Institute DSMZ-German Collection of Microorganisms and Cell Cultures (Braunschweig, Germany).

In all tests, the type strains *A. kunkeei* DSM 12361 and *L. rhamnosus* GG ATCC 53103 were used as controls.

### 2.2. Biochemical Characterization

*A. kunkeei* K18, K34 and K45 were assessed for their enzymatic and carbohydrate fermentation patterns using API ZYM system kit and API 50CHL system kit, respectively, according to manufacturer’s instructions (bioMérieux SA, Marcy l’Etoile, France). For comparative purposes, *A. kunkeei* DSM 12361 and *L. rhamnosus* GG ATCC 53103 were also tested.

### 2.3. Antibiotic Susceptibility

The antibiotic susceptibility of *A. kunkeei* K18, K34 and K45 was assessed with the Epsilometer test (E-test) gradient technology (Biomerieux, Marcy-l’Etoile, France) to determine the minimum inhibitory concentration (MIC) of various antimicrobial agents. For comparative purposes, *A. kunkeei* DSM 12361 and *L. rhamnosus* GG ATCC 53103 were also tested. In the present study, chloramphenicol, clindamycin, ampicillin, gentamicin, tetracycline, streptomycin, kanamycin and erythromycin were used in a concentration range of 0.016–256 µg/mL. The antibiotics were selected on the basis of the EFSA document regarding bacteria of human importance, and the cut-off values are those indicated in the same document [24]. BHI agar (Oxoid Ltd., Hampshire, UK) plates were inoculated with the bacterial suspensions in a sterile saline solution. The bacterial cell density of suspensions was adjusted to match McFarland turbidity standard 0.5 using a spectrophotometer (Bio-spectrometer Basic, Eppendorf, Italy).

After drying the plates surfaces for 15 to 20 min, the E-test strips of tested antibiotics were applied directly onto the surface. The plates were incubated overnight under anaerobic conditions at 37 °C; then, the MIC values were read following the manufacturer’s instructions.

### 2.4. Antimicrobial Activity

*A. kunkeei* K18, K34 and K45 (producers) were tested for their antimicrobial activity against the following indicator strains: *E. faecalis* ATCC 29212, *E. coli* ATCC 11775, *E. coli* ATCC 25404, *S. aureus* ATCC 29213 and *P. aeruginosa* ATCC 27853. For comparative purposes, *A. kunkeei* DSM 12361 and *L. rhamnosus* GG ATCC 53103 were also tested as producer strains.

The method used was the agar well diffusion assay following the protocol of Testa et al. (2019) [25] with some modifications. Briefly, 40 mL of BHI soft agar (0.7% agar) inoculated with an overnight culture of each indicator strain (final concentration of about 7 log CFU/mL) was poured into two Petri plates per each indicator strain. Three wells of 5.0 mm in diameter were bored into plates and 100 μL of an overnight broth culture (BC) of each producer strain w placed into a well. One well was used as control, housing 100 μL of uninoculated MRS broth acidified at pH 3.4 with HCl 1N (Sigma-Aldrich, Milan, Italy), that is, the lowest pH reached by indicator strains after incubation at 37 °C for 48 h. Plates were refrigerated at 4 °C for 4 h prior to incubation [26], which was subsequently performed at 37 °C for 24 to 48 h. After incubation, a calibrated densitometer (GS-800, BioRad, Hercules, CA, USA) was used for plate image acquisition, and Adobe Photoshop CC software was used for zone of inhibition (ZOI) measurement.

The well diffusion assay was also used to evaluate the antimicrobial activity of cell-free supernatants (CFSs) using the same procedure described above. CFSs were obtained by overnight cultures in MRS broth of each producer strain filter-sterilized (0.22 μm pore size, Schleider & Schuell, Dassel, Germany) before being disposed in the wells. Only zone diameters ≥ 8.0 mm were measured considering the lowest breakpoint value reported for resistance of the bacterial species used as indicators against different antibiotics (https://eucast.org/clinical_breakpoints/).

The experiment was carried out in triplicate and the antimicrobial activity was reported as diameter of ZOI ± SD (Standard Deviation) [27,28].

### 2.5. Osmotic Tolerance Assay in Sugar Syrup

Osmotic tolerance assay was performed in sugar syrup as reported by Iorizzo et al. (2020) [29]. Briefly, *A. kunkeei* K18, K34, K45 and DSM 12361 and *L. rhamnosus* GG ATCC 53103 grown overnight in MRS broth at 37 °C were harvested by centrifugation at 8000 rpm for 10 min at 4 °C. Pellets, washed twice with physiological solution, were inoculated in order to have an initial concentration of about 8 log CFU/mL. The experimental conditions were the following: batch A, sugar syrup composed of 40% glucose + 20% fructose (*w*/*v*) in distilled water at pH 4.2; batch B, sugar syrup composed of 40% glucose + 30% fructose (*w*/*v*) in distilled water at pH 4.2; batch C, sugar syrup composed of 50% sucrose (*w*/*v*) in distilled water at pH 4.2. The sugar syrups were acidified using HCl 1N and were sterilized by filtration (0.22 μm pore size-cellulose acetate filter). The cell viability was determined at time 0 and after 7 and 30 days of incubation at 4 and 20 °C, using MRS agar plates incubated at 37 °C for 48 h in anaerobic conditions.

### 2.6. Simulated Gastrointestinal Conditions

The survival during a simulated gastrointestinal transit (GIT) was evaluated following a protocol developed by reworking those described previously. [30,31,32,33]. The trial was performed on *A. kunkeei* K18, K34 and K45, while strains DSM 12361 and *L. rhamnosus* GG ATCC 53103 were assessed for comparative purposes. Each strain was grown overnight at 37 °C in 150 mL of MRS broth. Then cultures were centrifugated at 8000 rpm for 10 min at 4 °C (Centrifuge 5415 R; Eppendorf, Hamburg, Germany), and the pellets were resuspended in the same volume of sterile saline solution. Thirty-three milliliters of a sterile electrolyte solution simulating saliva (NaCl 6.2 g/L, KCl 2.2 g/L, CaCl_2_ 0.22 g/L and NaHCO_3_ 1.2 g/L) and lysozyme (Sigma-Aldrich) to a final concentration of 0.01% were added. After incubation for 2 min, microbial counts were performed by plate counts on MRS agar (Oxoid, Ltd. Hampshire, UK) placed at 37 °C for 72 h under anaerobic conditions (AnaeroGen, Oxoid Ltd., Hampshire, UK).

The solution containing each bacterium was then divided in six sterile bottles (portions of 30 mL each). To simulate the gastric environment, 5.5 mL of electrolyte solution containing 0.3% pepsin (final concentration) (Sigma-Aldrich) was added to each bottle. For each strain, two batches (consisting of three bottles each) were obtained: the pH was lowered to 2.0 in the former (batch pH 2), and it was lowered to 3.0 in the latter (batch pH 3) by adding HCl 1.0 N. Aliquots from each batch were collected after 30, 60 and 90 min and used for microbial counts performed as described above.

To simulate intestinal stress, oxygen was replaced by nitrogen in each bottle to obtain an anaerobic atmosphere, and the pH value was adjusted to 5.0 with a saturated sodium bicarbonate solution (8 g of sodium bicarbonate in 100 mL of distilled water, sterilized at 121 °C for 15 min). Then, 5.5 mL of a sterile electrolyte solution containing 0.45% porcine bile extract and 0.1% pancreatin (final concentration, both from Sigma-Aldrich) was added to each bottle. Thus, the pH was adjusted to 6.3 and slowly increased to 7.5 until the end of the intestinal stress test (4 h). Aliquots from each batch were collected and used for microbial counts carried out as previously described.

The solutions were freshly prepared daily and the whole study was performed at 37 °C.

### 2.7. Screening for Cholesterol Lowering

All chemical compounds described in this section were provided by Sigma-Aldrich (St. Louis, MO, USA). The method used was that described by Liong and Shah (2005) [34] with some modifications. Overnight cultures of *A. kunkeei* K18, K34 and K45 were inoculated (1%) into sterile MRS broth (Oxoid) supplemented with 0.2% (wt/vol) sodium thioglycolate and 0.3% (wt/vol) oxgall. Water-soluble cholesterol (cholesteryl-polyethylene glycol 600 sebacate) was filter-sterilized and added to the broth at a final concentration of 100 μg/mL. Cultures were anaerobically incubated for 24 h at 37 °C by using the GasPak Anaerobic System (Oxoid). After incubation, bacterial cells were removed by centrifugation (8000 rpm for 10 min at 4 °C). Cell-free supernatants (CFSs) were then assayed for their cholesterol content using an enzymatic kit (R-Biopharm, Milan, Italy) and a BioSpectrometer (Eppendorf, Hamburg, Germany). Strains *A. kunkeei* DSM 12361 and *L. rhamnosus* GG ATCC 53103 were assessed for comparative purposes, while uninoculated MRS broth was used as control.

### 2.8. Statistical Analysis

All data were expressed as mean ± standard deviation (SD) of three independent experiments. Statistical analysis was performed through the analysis of variance (ANOVA) followed by the Tukey’s multiple comparison. Statistical significance was attributed to *p* values < 0.05. The software SPSS (IBM SPSS Statistics 21) was used for the analysis.

## 3. Results

### 3.1. Biochemical Characterization

The results related to the enzymatic characterization of the LAB strains tested are shown in Table 1. All the strains of *A. kunkeei* had very similar enzymatic profiles; only strain DSM 12361 differed because it had no α-fucosidase activity. In addition, DSM 12361 did not show β-galactosidase activity, found in all the other tested strains. The β-glucosidase was absent in K45, whereas β-glucuronidase was detected only in the strain K34. *L. rhamnosus* GG ATCC 53103, used for comparative purposes considering its widespread use as probiotic, showed a markedly different enzymatic profile. In fact, it held lipase, trypsin, α-chymotrypsin and α-mannosidase activity, which were absent in *A. kunkeei* strains, and it had no N-acetyl-β-glucosaminidase activity, which was present in *A. kunkeei* strains.

With regard to the assimilation of carbohydrates, all the strains were able to ferment D-galactose, D-glucose, D-fructose, D-mannitol and D-trehalose (Table 2). The three strains of *A. kunkeei* isolated from bees showed very different carbohydrate assimilation patterns from that of the type strain *A. kunkeei* DSM 12361, which was able to ferment only the following carbohydrates: D-fructose, D-galactose, D-glucose, D-mannitol, D-melibiose, D-saccharose and D-trehalose. *L. rhamnosus* GG ATCC 53103 showed a carbohydrate assimilation pattern different from that of all *A. kunkeei* strains, and it was the only strain able to ferment D-arabinose, inositol and L-fucose.

### 3.2. Antibiotic Susceptibility and Antimicrobial Activity

Results of the susceptibility test against different antibiotic agents are reported in Table 3. Only *A. kunkeei* K45 was susceptible to all antibiotics tested, while the strain K34 showed resistance to three antibiotics (ampicillin, chloramphenicol and kanamycin). Strains K18 and DSM 12361 were resistant to only two antibiotics, that is, ampicillin for both, kanamycin for the former and chloramphenicol for the latter. *L. rhamnosus* GG ATCC 53103 was resistant only to kanamycin.

The results of the antimicrobial activity exerted by LAB against different indicator bacteria are reported in Table 4. In particular, the BC of *A. kunkeei* K18, K34 and K45 showed a ZOI of 12.4, 14.0 and 12.0 mm, respectively, against *P. aeruginosa* ATCC 27853; the CFS of K18 and K45 tested against the same indicator produced a ZOI of 11.0 mm in both cases, while the CFS of K34 gave a ZOI of 12.5 mm. The BC and CFS of *A. kunkeei* K34 showed a ZOI of 15.4 and 14.2 mm, respectively, against *E. faecalis* ATCC 2912. All other producers showed an antimicrobial activity with ZOI < 8.0 mm.

### 3.3. Bacterial Viability in Sugar Syrup

The results of *A. kunkeei* viability during 30 days of storage in sugar syrups are reported in Table 5. After 7 days of storage at 4 °C, the viability of tested strains remained almost unchanged in batch A (40% glucose + 20% fructose). Instead, at 20 °C, a charge reduction between 3 log (K18) and 6 log (DSM 12361) was observed. After 30 days of storage at 4 °C, the survival of all bacteria was still high. In particular, the strain K18 maintained almost the same initial charge, while at 20 °C all the strains were no longer detectable.

As for batch B (40% glucose + 30% fructose), after 7 days of storage at 4 °C, viable cells decreased by about 1 log; after 30 days of storage, viable cells decreased by about 3 log. In batch B stored at 20 °C, a limited survival (1.68 log CFU/mL) was found only for the strain K18 after 7 days of storage, while the other strains were no longer detectable. In batch C (50% sucrose), the viability of all the strains remained substantially stable after 7 days of storage at 4 °C; after 30 days at 4 °C, less than 1 log reduction was observed for all bacteria. After 7 days of storage at 20 °C, there was a decrease in viable counts by about 1–2 logs, with the decrease after 30 days reaching about 4 logs for strains K18, K34 and K45 and 5 logs for the strain DSM 12361. The strain *L. rhamnosus* GG ATCC 53103 showed much lower viabilities than those recognized for all the strains of *A. kunkeei* tested, independent of the batch, storage time and temperature.

### 3.4. Viability during Simulated Gastrointestinal Transit

**Figure 1:**
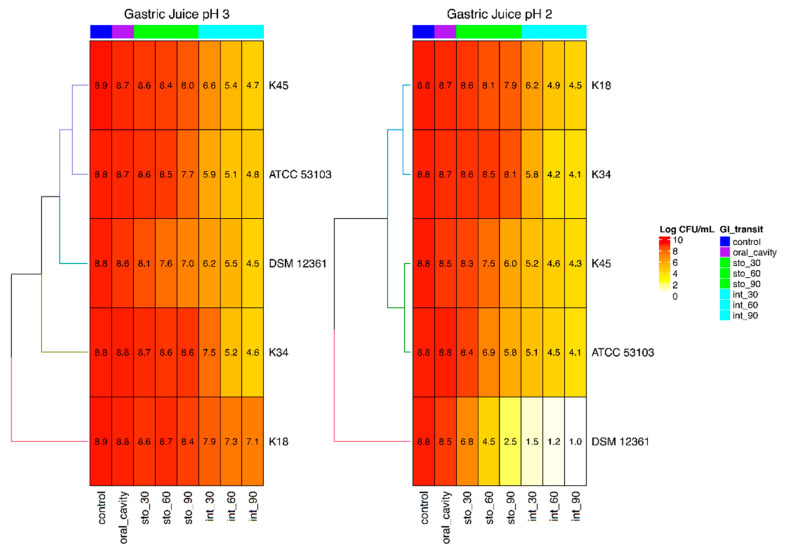
Heat maps depicting bacterial viability of *A. kunkeei* K18, K34, K45 and DSM 12361 and *L. rhamnosus* GG ATCC 53103 in simulated gastrointestinal conditions.

The in vitro effect of gastrointestinal conditions on the viability of tested strains is summarized in Figure 1. Simulation of oral cavity conditions did not produce relevant changes in counts for all tested bacteria. Counts changed only slightly after 90 min in simulated gastric juice at pH 3.0 for strains K18, K34 and K45, while strains ATCC 53103 and DSM 12361 decreased by approximately 1 and 2 log, respectively. As expected, lower permanence times (30 and 60 min) in simulated stomach conditions at pH 3.0 gave higher counts than those recorded after 90 min. All the strains were subjected to the intestinal tract simulation after 30, 60 and 90 min of permanence in gastric juice at pH 3.0. At the end of the trial, the lowest counts were detected for strains that were previously left for 90 min in simulated gastric conditions. However, significant differences were highlighted after the intestinal simulated transit. In fact, among strains retained 90 min in simulated gastric juice, *A. kunkeei* K18 showed the highest vitality (7.10 log CFU/mL), with a decrease of about 1.8 log compared to the initial count. A high loss of viability was detected for all the other tested strains, for which final counts between 4.8 and 4.5 log CFU/mL were registered.

The simulated transit in the stomach at pH 2.0, especially for 90 min, severely affected the survival of some tested strains, such as *A. kunkeei* DSM 12361, which showed more than 4 log difference in survival when compared to 90 min of gastric simulation at pH 3.0 and final counts < 1.0 log CFU/mL after intestinal simulation. Among the other strains, *A. kunkeei* K45 and *L. rhamnosus* GG ATCC 53103 were those that mainly suffered from the simulated passage in the stomach at pH 2.0 for 90 min (2 and 1.85 log difference in survival when compared 90 min of gastric simulation at pH 3.0, respectively), whereas the strain *A. kunkeei* K18 seemed to be more susceptible to the simulated intestinal environment, especially after 60 and 90 min of permanence in simulated gastric juice at pH 2.0. However, K18 tested at pH 2.0 showed the best performance, as evidenced by final counts of 4.5 log CFU/mL.

### 3.5. Cholesterol Lowering Capacity

Table 6 shows the results of the cholesterol assimilation test. *A. kunkeei* K18 and *L. rhamnosus* GG ATCC 53103 assimilated, after 24 h, more than 50% of the initial cholesterol. *A. kunkeei* K45 and DSM 12361 showed a lower ability to assimilate cholesterol (about 20%). The strain K34 showed no ability to lower cholesterol.

## 4. Discussion

*A. kunkeei* is microorganism that is considered to be able to protect honeybees from different pathogens, suggesting its beneficial role for the health of these insects, where it is frequently found in the gut [29,35]. In the last few years, strains belonging to this species were also investigated for their possible positive effect on the human health. For instance, Asama et al. (2016) [36] highlighted that heat-killed *A. kunkeei* strains could exert a beneficial role in the human intestinal tract mainly through a possible indirect mechanism involving the modulation of epithelial-derived antimicrobials able to affect the colonization of *Bacteroides* spp. Actually, some authors pointed out in a recent review that the use of heat-killed probiotics, cell-free supernatants and purified compounds is able to confer beneficial effects, mainly through the release of bacterial components with key immunomodulating effects and antagonizing properties against pathogens [37].

Other authors highlighted the beneficial role of live bacteria for human health. In this context, Sakandar et al. (2019) [8] showed that *A. kunkeei*, as well as other fructophilic lactic acid bacteria (FLAB), are able to readily utilize fructose in cereal fermentation and in the baking industry, helping in the prevention of irritable bowel syndrome (IBS), since fructose malabsorption is considered as an adverse factor in this condition. The ability to utilize fructose by *A. kunkeei* was confirmed in our study, and it represents a distinctive feature of FLAB. In fact, these bacteria, differently from other lactic acid bacteria (LAB), produce from fructose very reduced levels of ethanol under anaerobic conditions, while they form high levels of mannitol from fructose to regenerate NAD^+^, thus growing quickly in substrates where fructose is present [38]. Noteworthily, mannitol produced by strains of *A. kunkeei* may represent another positive tract of probiotic interest, since this polyol is considered as a prebiotic with protective effects on probiotic bacteria [32]. As for the other carbohydrates tested in our study, *A. kunkeei* showed composite fermentation profiles, as also reported by other authors [18,39,40]. In fact, strains K18, K34 and K45 were able to metabolize between 20 and 28 out of 49 carbohydrates tested, similar to if not more than those used by *L. rhamnosus* GG ATCC 53103 (22 in total), included as control, and much more than those fermented by the type strain *A. kunkeei* DSM 12361 (only 7). Our data agree with those highlighted in other studies with regard to the fermentative profile of the type strain *A. kunkeei* DSM 12361 [41,42] but are in contrast with those recorded for other *A. kunkeei* strains, which showed a limited fermentation of carbohydrates or a fermentation profile different from that detected in our study [18].

All tested *A. kunkeei* strains presented similar enzymatic profiles except as regards β-glucosidase, which was undetectable in strain K45; α-fucosidase and β-galactosidase, unavailable only in *A. kunkeei* DSM 12361; and β-glucuronidase, which was present only in K34. Among tested enzymes, β-glucosidase, β-galactosidase and leucine arylamidase are known as beneficial ones, since they are involved in the hydrolysis of glycosidic bonds, lactase and protease, respectively [43]. The three tested strains of *A. kunkeei* produced leucine arylamidase and β-galactosidase, whereas β-glucosidase was absent only in strain K45. Among other beneficial enzymes, the presence of α-fucosidase in tested strains is particularly interesting from a probiotic point of view. In fact, this enzyme indicates the ability to utilize certain fucosyloligosaccharides present in the infant and adult human gut mucosa. Therefore, this enzyme can be of great importance for the microbial survival in such a complex habitat, where these oligosaccharides may represent one of the most important carbon and energy sources [44].

With regard to the presence of other enzymes, N-acetyl-β-glucosaminidase, α-chymotrypsin and β-glucuronidase are considered as harmful ones that should be absent in probiotics. These enzymes are associated with the production of toxic, mutagenic and carcinogenic products involved in the development of gastrointestinal (GI) diseases [45]. In this context, tested *A. kunkeei* strains were positive for N-acetyl-β-glucosaminidase, with only K34 being positive for β-glucuronidase, but they were negative for α- chymotrypsin. Noteworthily, the probiotic strain *L. rhamnosus* GG ATCC 53103, used as control, resulted as positive for chymotrypsin.

The evaluation of the possible role of *A. kunkeei* strains as probiotics proceeded with the ascertainment of their sensitivity to different antibiotics, considering that the marketing of antibiotic-resistant probiotics could represent a high risk due to the horizontal spread of resistance, with the possibility of being transferred to intestinal pathogens [46]. For this reason, these features should always be measured for safety reasons in potentially beneficial microorganisms prior to their use as probiotics. Therefore, in this study, the strains of *A. kunkeei* were tested against various antibiotics following the EFSA suggestions regarding bacteria of human importance [24,36]. Only strains K34 and DSM 12361 showed resistance to chloramphenicol, in accordance with the study of the genome of one strain of *A. kunkeei* isolated from a Chilean honeybee gut by Olmos et al. (2014) [47]. Specifically, they found a chloramphenicol acetyltransferase, an enzyme responsible for chloramphenicol resistance. Three strains, i.e., K18, K34 and DSM 12361, were found to be resistant to ampicillin, and K18 and K34 were also resistant to kanamycin, which was the sole antibiotic unable to inhibit *L. rhamnosus* GG ATCC 53103. Antibiotic resistances observed for *A. kunkeei* in our study are different from those reported by Ebrahimi et al. (2020) [48] in a recent research article. Specifically, these authors found a high resistance to several antibiotics tested in their study, including five drugs also evaluated in our investigation. Basically, this fact can be explained by a strain-dependent behavior, as reported previously [49], but it is important to remember that the antibiotic resistance can be intrinsic or acquired, and the acquisition, in turn, may result from a chromosomal mutation or horizontal gene transfer by means of transposons, plasmids and integrons [50,51]. According to some authors who have studied the genome of *A. kunkeei*, the antibiotic resistance is encoded by a single and stable chromosomal gene, and horizontal gene transfer mechanisms between members of the honeybee gut microbiota were not detected [47,52,53].

The antimicrobial activity of tested strains was evaluated against some foodborne pathogens or spoilage bacteria, such as *S. aureus*, *E. coli*, *P. aeruginosa* and *E. faecalis*, with the last one being frequently involved in nosocomial infections. A risk linked to the access in the gut of these harmful bacteria is related to their potential translocation in the bloodstream, causing infections such as bacteremia, peritonitis and endocarditis [54]. This phenomenon is particularly dangerous in immunocompromised patients due to the possible onset of systemic infections. In such circumstances, the administration of appropriate antibacterial agents represents the best choice even today, but some of these pathogens are able to form biofilm, thus protecting themselves from antibiotics through the production of extracellular substances [55]. Several antimicrobial agents other than antibiotics are able to reach microorganisms protected by their own external matrix. In this field, interesting results were obtained with the use of natural antimicrobials, such as phenyllactic acid produced by lactic acid bacteria against *Listeria* spp. [28] or biofilm produced by *A. kunkeei* and able to mitigate infections by *P. aeruginosa* [21]. Moreover, in a recent work, it was shown that some LAB, including strains of *A. kunkeei*, used in a honey-based gelatinous matrix had antagonistic activity against pathogens most commonly involved in chronic wounds. A topical treatment was proposed as an alternative or antibiotic support solution [19]. In line with these results, we also found an interesting anti-*Pseudomonas* effect exerted by broth cultures and cell-free supernatants of tested *A. kunkeei* strains. Moreover, one of them, labelled as K34, was also able to inhibit *E. faecalis*. Considering that in our study no effect on the growth of indicators was detected by using an acidified control medium, the inhibition exerted by some *A. kunkeei* strains against *P. aeruginosa* and *E. faecalis* cannot be ascribed to a mere consequence of the pH lowering due to producer strain metabolism. Instead, the antimicrobial activity was most likely due to the production of organic acids, proteinaceous compounds or other antimicrobial metabolites by *A. kunkeei*, with these products persisting also in the cell-free supernatants, as already detected in previous studies involving *A. kunkeei* or other *Lactobacillus* species [26,48].

The ability of probiotic bacteria to reduce serum cholesterol levels was reported by several authors. Wang et al. (2018) [56] analyzed 32 randomized controlled trials and concluded that probiotics can significantly reduce serum total cholesterol. A recent review [57] considered 34 different studies published since 1990, and a significant correlation between probiotic intake and alleviation of cardiovascular disease risk factors, including high cholesterol, was established. Actually, the in vitro screening for cholesterol-lowering properties has become an important criterion in the selection of bacterial strains prior to their use in vivo, since the presence of such properties in vitro can be used as a predictor for in vivo cholesterol-lowering activity [58].

Our results revealed that some strains of *A. kunkeei* are able to assimilate cholesterol, confirming what has reported in other studies [8]. Notably, the strain K18 was able to lower the cholesterol concentration by more than 50%. This amount was similar to that registered for the control strain *L. rhamnosus* GG ATCC 53103 and comparable to that reported for other LAB in similar conditions [59]. Several possible mechanisms for cholesterol removal by probiotics have been proposed during recent years, including assimilation of cholesterol by growing cells, binding of cholesterol to cellular surface, incorporation of cholesterol into the cellular membrane, deconjugation of bile via bile salt hydrolase and coprecipitation of cholesterol with deconjugated bile; however, as also highlighted in our work, some of these mechanisms are strain-dependent [60,61,62], and there is a need for further investigations to understand the pathways that determine the cholesterol lowering [63].

Considering that most of the positive effects are expressed in the gut, where probiotics arrive via ingestion of formulations or foods containing an adequate number of microorganisms, the screening of resistance to gastrointestinal stressors represents an important selection criterion even today. In fact, it is well known that the passage through the GI tract can negatively affect the vitality and functionality of beneficial microorganisms [30,32]. Apart from the type strain DSM 12361, the results obtained herein highlighted for tested strains of *A. kunkeei* a survival rate equal to if not greater than that showed by *L. rhamnosus* GG ATCC 53103, used as probiotic control. This is an important result, considering that our previous findings showed a strong reduction in viable counts after GI stress for different bacterial strains marketed as probiotics [30].

Finally, some of the tested strains of *A. kunkeei* exhibited an excellent viability in presence of high sugar concentrations, especially *A. kunkeei* K18 stored at 4 °C for 30 days in 40% glucose + 20% fructose and in 50% sucrose. This last datum could be of particular interest because it demonstrates that selected strains of *A. kunkeei* can be used for the probiotication of fruit preparations (mousses, jams, jellies, marmalades and juices), which are often used in the diet of hospitalized and immunocompromised patients, especially, in light of previous results, to strengthen their defenses against *P. aeruginosa* and *E. faecalis*.

Other studies demonstrated the feasibility of using probiotic LAB in fruit products [64,65,66]. However, their low survival and stability in such matrices were major drawbacks. In light of our results, it is reasonable to assume that the osmotolerance of *A. kunkeei* is linked to the ability of this species to survive in stressful conditions, such as fructose-rich niches. Obviously, further studies are required to confirm the viability of tested strains in fruit-based foods and to evaluate their impact on chemical and sensorial features of obtained products.

## 5. Conclusions

In summary, strains of *A. kunkeei* tested in our study showed a number of positive features that match with those required for probiotic bacteria. The strain K18, in particular, displayed the most promising functional properties, but others showed performances similar to if not better than those detected in *L. rhamnosus* GG, used as probiotic control in each trial.

In conclusion, this work provides evidence on the opportunity to exploit honeybees as relatively unexplored sources for the isolation of novel LAB strains for their potential use as probiotics in humans.

## Figures and Tables

**Table 1:** Enzymatic profiles of *A. kunkeei* K18, K34, K45 and DSM 12361 and *L. rhamnosus* GG ATCC 53103 detected through API ZYM kit.

	K18	K34	K45	DSM 12361	ATCC 53103
Acid phosphatase	+	+	+	+	+
Alkaline phosphatase	+	+	+	+	+
Cystine arylamidase	+	+	+	+	+
Esterase (C4)	+	+	+	+	+
Esterase lipase (C8)	+	+	+	+	+
Leucine arylamidase	+	+	+	+	+
Lipase (C14)	-	-	-	-	+
N-Acetyl-β-glucosaminidase	+	+	+	+	-
Naphthol-AS-BI-phosphohydrolase	+	+	+	+	+
Trypsin	-	-	-	-	+
Valine arylamidase	+	+	+	+	+
α-Chymotrypsin	-	-	-	-	+
α-Fucosidase	+	+	+	-	+
α-Galactosidase	-	-	-	-	-
α-Glucosidase	+	+	+	+	+
α-Mannosidase	-	-	-	-	+
β-Galactosidase	+	+	+	-	+
β-Glucosidase	+	+	-	+	+
β-Glucuronidase	-	+	-	-	-

**Table 2:** Carbohydrates fermented by *A. kunkeei* K18, K34, K45 and DSM 12361 and *L. rhamnosus* GG ATCC 53103 detected through API 50 CH kit.

	K18	K34	K45	DSM 12361	ATCC 53103
Amidon	-	-	-	-	-
Amygdaline	+	+	-	-	+
Arbutine	+	+	+	-	+
D-Adonitol	-	-	-	-	-
D-Arabinose	-	-	-	-	+
D-Arabitol	-	-	-	-	-
D-Cellobiose	+	+	+	-	+
D-Fructose	+	+	+	+	+
D-Fucose	-	-	-	-	-
D-Galactose	+	+	+	+	+
D-Glucose	+	+	+	+	+
D-Lactose	+	+	+	-	-
D-Lyxose	-	-	-	-	-
D-Maltose	+	+	+	-	-
D-Mannitol	+	+	+	+	+
D-Mannose	+	+	+	-	+
D-Melezitose	-	-	+	-	+
D-Melibiose	-	+	+	+	-
D-Raffinose	-	-	+	-	-
D-Ribose	+	+	+	-	+
D-Saccharose	+	+	+	+	-
D-Sorbitol	-	+	+	-	+
D-Tagatose	-	-	+	-	+
D-Trehalose	+	+	+	+	+
D-Turanose	+	+	+	-	-
Dulcitol	-	-	+	-	+
D-Xylose	-	-	+	-	-
Erythritol	-	-	-	-	-
Esculine	+	+	+	-	+
Gentiobiose	+	+	+	-	+
Glycerol	-	-	-	-	-
Glycogene	-	-	-	-	-
Inositol	-	-	-	-	+
Inuline	-	-	-	-	-
L-arabinose	+	+	+	-	-
L-Arabitol	-	-	-	-	-
L-Fucose	-	-	-	-	+
L-Rhamnose	-	-	-	-	-
L-Sorbose	-	-	+	-	-
L-Xylose	-	-	+	-	-
Methyl-a-D-Glucopyranoside	-	-	-	-	-
Methyl-a-D-Mannopyranoside	-	+	+	-	-
Methyl-b-D-Xylopyranoside	-	+	-	-	-
N-Acetyl-Glucopyranoside	+	+	+	-	+
Potassium 2-Cetogluconate	-	-	-	-	-
Potassium 5-Cetogluconate	+	+	-	-	-
Potassium Gluconate	-	-	+	-	+
Salicine	+	+	-	-	+
Xylitol	-	-	-	-	-

**Table 3:** Antibiotic susceptibility of *A. Kunkeei* K18, K34, K45 and DSM 12361 and *L. rhamnosus* ATCC 53103 as determined with E-test. Cut-off values are expressed in µg/mL. Resistance is reported in bold.

Antibiotics	Strains						
	Cut-Off *L. rhamnosus* *	Cut-Off Lactobacillus Obligate Heterofermentative *	K18	K34	K45	DSM 12361	ATCC 53103
Ampicillin	4	2	**R**	**R**	S	**R**	S
Chloramphenicol	4	4	S	**R**	S	**R**	S
Clindamycin	1	1	S	S	S	S	S
Erythromycin	1	1	S	S	S	S	S
Gentamycin	16	16	S	S	S	S	S
Kanamycin	64	32	**R**	**R**	S	S	**R**
Streptomycin	32	64	S	S	S	S	S
Tetracycline	8	8	S	S	S	S	S

* EFSA, 2012 [24].

**Table 4:** Antimicrobial activity exerted by broth cultures (BCs) and cell-free supernatants (CFSs) of *A. kunkeei* K18, K34, K45 and DSM 12361 and *L. rhamnosus* GG ATCC 53103 (producers) against *S. aureus* ATCC 29213, *E. faecalis* ATCC 29212, *E. coli* ATCC 11775, *E. coli* ATCC 25404 and *P. aeruginosa* ATCC 27853 (indicators). Only zones of inhibition (ZOIs) ≥ 8.0 mm are reported.

Indicator Strains	Zone Diameter Breakpoints (R<) *	Producer Strains (Broth Cultures, BC; Cell-Free Supernatants, CFS)						
			K18	K34	K45	DSM 12361	ATCC 53103	MRS pH 3.4
*S. aureus* ATCC 29213	17.0–26.0	BC	-	-	-	-	-	-
		CFS	-	-	-	-	-	-
*E. faecalis* ATCC 29212	8.0–22.0	BC	-	15.4 ± 0.2	-	-	-	-
		CFS	-	14.2 ± 0.3	-	-	-	-
*E. coli* ATCC 11775	12.0–25.0	BC	-	-	-	-	-	-
		CFS	-	-	-	-	-	-
*E. coli* ATCC 25404	12.0–25.0	BC	-	-	-	-	-	-
		CFS	-	-	-	-	-	-
*P. aeruginosa* ATCC 27853	15.0–26.0	BC	12.4 ± 0.2	14.0 ± 0.1	12.0 ± 0.2	-	-	-
		CFS	11.0 ± 0.1	12.5 ± 0.2	11.0 ± 0.1	-	-	-

* Intervals of zone diameter breakpoints (mm) reported for resistance (R<) of the bacterial species used as indicators against different antibiotics (available at https://eucast.org/clinical_breakpoints/).

**Table 5:** Survival of *A. kunkeei* K18, K34, K45 and DSM 12361 and *L. rhamnosus* GG ATCC 53103 in different sugar syrups (A: 40% glucose + 20% fructose; B: 40% glucose + 30% fructose; C: 50% sucrose) after 0, 7 and 30 days of storage at 4 and 20 °C. Values represent the average (±SD) of three biological replicates.

Storage Time (days)	Sugar Syrup (pH 4.2)	Viability (log CFU/mL)									
		4 °C					20 °C				
		K18	K34	K45	DSM 12361	ATCC 53103	K18	K34	K45	DSM 12361	ATCC 53103
T_0_	A 40% glucose 20% fructose	8.31 ^Aa^ ± 0.04	8.09 ^Ab^ ± 0.05	8.32 ^Ac^ ± 0.07	8.24 ^Ab^ ± 0.06	8.12 ^Ac^ ± 0.05	8.65 ^Cc^ ± 0.03	8.20 ^Ac^ ± 0.05	8.42 ^Bc^ ± 0.02	8.57 ^Cc^ ± 0.01	8.34 ^Bb^ ± 0.05
T_7_		8.23 ^Ba^ ± 0.05	8.00 ^Bb^ ± 0.03	8.11 ^Bb^ ± 0.07	8.19 ^Bb^ ± 0.04	7.12 ^Ab^ ± 0.06	5.23 ^Eb^ ± 0.05	4.30 ^Db^ ± 0.02	3.21 ^Cb^ ± 0.03	2.60 ^Bb^ ± 0.02	<1 ^Aa^
T_30_		8.18 ^Da^ ± 0.07	7.72 ^Ca^ ± 0.06	7.73 ^Ca^ ± 0.03	7.44 ^Ba^ ± 0.04	3.43 ^Aa^ ± 0.03	<1 ^Aa^	<1 ^Aa^	<1 ^Aa^	<1 ^Aa^	<1 ^Aa^
T_0_	B 40% glucose 30% fructose	8.63 ^Bc^ ± 0.04	8.19 ^Ac^ ± 0.02	8.22 ^Ac^ ± 0.03	8.14 ^Ac^ ± 0.04	8.18 ^Ac^ ± 0.03	8.72 ^Cc^ ± 0.06	8.35 ^Ab^ ±0.03	8.43 ^Bb^ ± 0.05	8.35 ^Ab^ ± 0.04	8.32 ^Ab^ ± 0.07
T_7_		7.87 ^Db^ ± 0.06	7.30 ^Cb^ ± 0.05	7.11 ^Bb^ ± 0.04	7.09 ^Bb^ ± 0.03	5.34 ^Ab^ ± 0.04	1.68 ^Bb^ ± 0.01	<1 ^Aa^	<1 ^Aa^	<1 ^Aa^	<1 ^Aa^
T_30_		5.98 ^Da^ ± 0.04	5.72 ^Ca^ ± 0.03	5.73 ^Ca^ ± 0.04	4.44 ^Ba^ ± 0.03	1.20 ^Aa^ ± 0.03	<1 ^Aa^	<1 ^Aa^	<1 ^Aa^	<1 ^Aa^	<1 ^Aa^
T_0_	C 50% sucrose	8.23 ^Ac^ ± 0.01	8.15 ^Ac^ ± 0.03	8.28 ^Ac^ ± 0.04	8.20 ^Ac^ ± 0.02	8.23 ^Ac^ ± 0.07	8.71 ^Dc^ ± 0.03	8.25 ^Ac^ ± 0.02	8.62 ^Cc^ ± 0.05	8.44 ^Bc^ ± 0.02	8.23 ^Ac^ ± 0.03
T_7_		8.12 ^Bb^ ± 0.06	8.06 ^Bb^ ± 0.01	8.00 ^Bb^ ± 0.03	8.10 ^Bb^ ± 0.02	7.12 ^Ab^ ± 0.05	7.15 ^Db^ ± 0.05	6.95 ^Cb^ ± 0.04	6.80 ^Bb^ ± 0.03	6.89 ^Cb^ ± 0.02	5.50 ^Ab^ ± 0.03
T_30_		7.90 ^Da^ ± 0.06	7.55 ^Ba^ ± 0.03	7.50 ^Ba^ ± 0.05	7.65 ^Ca^ ± 0.04	4.23 ^Aa^ ± 0.04	4.63 ^Ea^ ± 0.02	4.30 ^Da^ ± 0.03	4.21 ^Ca^ ± 0.02	3.60 ^Ba^ ± 0.03	<1 ^Aa^

For each sugar syrup and temperature condition, different lowercase letters in each column, and uppercase letters in each row, represent significant differences (*p* < 0.05). Results are shown as mean ± standard deviation (*n* = 3).

**Table 6:** Cholesterol amounts recovered in cell-free supernatants from broth cultures (in MRS) inoculated with *A. kunkeei* K18, K34, K45 and DSM 12361 and *L. rhamnosus* GG ATCC 53103 and analyzed after 24 h of incubation at 37 °C. Control, uninoculated MRS broth.

	Control	K18	K34	K45	DSM 12361	ATCC 53103
Cholesterol (μg/mL)	10.03 ± 0.35	4.12 ± 0.50	10.02 ± 0.60	8.24 ± 0.44	7.90 ± 0.40	4.02 ± 0.43
